# Performance Assessment of the Mortality in Emergency Department Sepsis Score, Modified Early Warning Score, Rapid Emergency Medicine Score, and Rapid Acute Physiology Score in Predicting Survival Outcomes of Adult Renal Abscess Patients in the Emergency Department

**DOI:** 10.1155/2018/6983568

**Published:** 2018-09-19

**Authors:** Su-Han Chang, Chiao-Hsuan Hsieh, Yi-Ming Weng, Ming-Shun Hsieh, Zhong Ning Leonard Goh, Hsien-Yi Chen, Tung Chang, Chip-Jin Ng, Joanna Chen-Yeen Seak, Chen-Ken Seak, Chen-June Seak

**Affiliations:** ^1^Department of Emergency Medicine, Lin-Kou Medical Center, Chang Gung Memorial Hospital, Taoyuan, Taiwan; ^2^Department of Emergency Medicine, Prehospital Care Division, Taoyuan General Hospital, Ministry of Health and Welfare, Taoyuan, Taiwan; ^3^Department of Emergency Medicine, Taipei Veterans General Hospital, Taoyuan Branch, Taoyuan, Taiwan; ^4^School of Medicine, National Yang-Ming University, Taipei, Taiwan; ^5^Institute of Occupational Medicine and Industrial Hygiene, College of Public Health, National Taiwan University, Taipei, Taiwan; ^6^School of Medicine, International Medical University, Kuala Lumpur, Malaysia; ^7^College of Medicine, Chang Gung University, Taoyuan, Taiwan; ^8^Sarawak General Hospital, Kuching, Sarawak, Malaysia

## Abstract

**Background:**

Renal abscess is a relatively uncommon yet debilitating and potentially fatal disease. There is no clearly defined, objective risk stratification tool available for emergency physicians' and surgeons' use in the emergency department (ED) to quickly determine the appropriate management strategy for these patients, despite early intervention having a beneficial impact on survival outcomes.

**Objective:**

This case control study evaluates the performance of Mortality in Emergency Department Sepsis Score (MEDS), Modified Early Warning Score (MEWS), Rapid Emergency Medicine Score (REMS), and Rapid Acute Physiology Score (RAPS) in predicting risk of mortality in ED adult patients with renal abscess. This will help emergency physicians, surgeons, and intensivists expedite the time-sensitive decision-making process.

**Methods:**

Data from 152 adult patients admitted to the EDs of two training and research hospitals who had undergone a contrast-enhanced computed tomography scan of the abdomen and was diagnosed with renal abscess from January 2011 to December 2015 were analyzed, with the corresponding MEDS, MEWS, REMS, RAPS, and mortality risks calculated. Ability to predict patient mortality was assessed via receiver operating curve analysis and calibration analysis.

**Results:**

MEDS was found to be the best performing physiologic scoring system, with sensitivity, specificity, and accuracy of 87.50%, 88.89%, and 88.82%, respectively. Area under receiver operating characteristic curve (AUROC) value was 0.9440, and negative predictive value was 99.22% with a cutoff of 9 points.

**Conclusion:**

Our study is the largest of its kind in examining ED patients with renal abscess. MEDS has been demonstrated to be superior to MEWS, REMS, and RAPS in predicting mortality for this patient population. We recommend its use for evaluation of disease severity and risk stratification in these patients, to expedite identification of critically ill patients requiring urgent intervention.

## 1. Introduction

Renal abscess, while being relatively uncommon at an incidence of 1 to 10 in 10,000 hospital admissions [1–3], is a debilitating and potentially fatal disease with mortality rates historically ranging from 12% to 50% [2, 4]. With the advent of ultrasonography and computed tomography imaging techniques enabling the early identification of patients with renal abscess and subsequent prompt intervention, survival rates have improved; nevertheless, mortality still reaches up to 8.3% currently [3, 5, 6].

Management of renal abscess comprises antimicrobial therapy, percutaneous drainage, surgical drainage, and nephrectomy [2, 4, 7]. Though surgical drainage and nephrectomy are often avoided as first-line interventions, Meng et al. (2002) demonstrated that 35% of patients ultimately required exploratory laparotomy and nephrectomy while Coelho et al. (2007) found that 18.5% and 23.1% of renal abscess patients had received surgical drainage and nephrectomy, respectively [4, 5]. Apart from being the last-ditch intervention after failure of medical therapy, nephrectomy is also indicated for renal abscesses which develop in small, scarred, poorly functioning kidneys [8]; however, it is almost impossible to establish this history in the emergency department (ED) setting as patients with such a condition usually present in a critical state, complicating the time-sensitive decision-making process for emergency physicians and surgeons.

Early appropriate intervention improves the survival outcome of each renal abscess patient; therefore, it is imperative that they are initiated on the right therapy in the ED immediately after accurate assessment of disease severity and the corresponding mortality risk. There is however yet a clearly defined, objective risk stratification tool that lays down the criteria which justifies the decision of choosing medical therapy over surgical intervention and vice versa.

Various studies have suggested that the prognosis of renal abscess patients is related to certain variables such as the presence of lethargy, elevated blood urea nitrogen, thrombocytopenia, abscess size, and serum C-reactive protein levels [6, 9, 10]. These variables nevertheless do not constitute a complete risk stratification tool, and thus we employed four already-established ED scoring systems and studied their performance in predicting the mortality risk of renal abscess patients in the ED.

The four chosen physiologic scoring systems are the more commonly used ones: Rapid Acute Physiology Score (RAPS) [11], Rapid Emergency Medicine Score (REMS) [12, 13], Modified Early Warning Score (MEWS) [14], and Mortality in Emergency Department Sepsis Score (MEDS) [15, 16]. These systems are comprised of easily and rapidly obtainable parameters that can be calculated by the bedside, allowing emergency physicians, surgeons, and intensivists to quickly and accurately identify critically ill patients in whom urgent intervention is necessitated.

This study is a continuation in our series of investigations into the use of scoring systems for risk stratification of patients with abdominal infections to improve patient outcomes. Our first study evaluated the performance of Simplified Acute Physiology Score II (SAPS II), the Acute Physiology and Chronic Health Evaluation II (APACHE II) score, and the Sequential Organ Failure Assessment (SOFA) score in predicting mortality of adult hepatic portal venous gas (HPVG) patients presenting to the ED and found that SAPS II performed the best [17]. We however realised that SAPS II, a score devised for intensive care units (ICUs), was not optimised for all ED environments. This was because some parameters of SAPS II required results of various investigations, readily available in the ICU, but cumbersome or not possible in some ED settings.

As such, we undertook a second study using ED scoring systems which incorporated parameters based on rapidly obtainable vital signs, while concurrently expanding our recruitment of adult ED HPVG patients to span 16.5 years. REMS was shown to outperform RAPS and MEWS [18].

Our third study was embarked upon to determine if there was any value in consideration of patient characteristics on top of clinical parameters, and if the results derived from the HPVG patient population could be similarly applied to splenic abscess ED patients. Statistical analysis results demonstrated that MEDS was superior to MEWS, REMS, and RAPS, thus giving merit to the hypothesis that patient characteristics should also be considered in patients with abdominal infections. We were however surprised to note that the performance of REMS in predicting mortality of splenic abscess patients was ranked last [19].

This current study was conceived with three aims: first, to evaluate the suitability of these four aforementioned ED scoring systems in patients with renal abscess, an abdominal infection with differing aetiology and pathogenesis from HPVG and splenic abscess; second, to ascertain the superiority of MEDS in the ED renal abscess patient population as a risk stratification tool which incorporates clinical parameters with patient characteristics; third, to assess the next-best score amongst RAPS, REMS, and MEWS to employ. MEDS's strength of taking into account patient characteristics is a potential weakness if past history cannot be adequately established quickly. This could be due to neurological deficits of the patient, lack of accompanying family members, or language barriers, amongst other issues, Plan B is required in such cases.

## 2. Materials and Methods

### 2.1. Study Design

This retrospective analysis was conducted at the EDs of two training and research hospitals, Linkou Chang Gung Memorial Hospital (3406 beds with approximately 17000 monthly ED visits in 2017) and Taipei Chang Gung Memorial Hospital (252 beds with approximately 4200 monthly ED visits in 2017). The Chang Gung Memorial Hospital Institutional Review Board approved this study (IRB: 201701502B0C501), waiving the need for consent from study participants. Data was accessed anonymously.

### 2.2. Settings and Subjects

All adult patients above the age of 18 years admitted to the ED who had undergone a contrast-enhanced computed tomography scan of the abdomen and was diagnosed with renal abscess from January 2011 to December 2015 were recruited. Those with concomitant abdominal infections were excluded from the study.

### 2.3. Criteria of Renal Abscess

The diagnosis of renal abscess was confirmed upon meeting at least 1 of the following criteria: (1) intraoperative findings of renal abscess; (2) histopathological evidence of renal abscess; and in the absence of surgical intervention, (3) positive clinical, laboratory, and imaging findings consistent with the diagnosis.

### 2.4. Measurement of Variables

Pertinent data was retrieved from the ED records of the identified patients and used to calculate the respective physiological scoring systems (Tables 1–4).* Septic shock* was defined in accordance with the Second International Consensus Definitions for Sepsis and Septic Shock criteria (2001) [20]. Patient mortality or survival upon discharge were taken as study endpoints.

### 2.5. Statistical Analysis

Median and interquartiles of numerical variables, as well as frequencies and corresponding percentages (%) of categorial variables, were recorded. Univariate analyses were conducted to establish the relationship between predictors and mortality rates; Mann–Whitney* U* tests and Fisher's exact tests were selected for such analyses for numerical and categorical variables, respectively, in view of the small nonsurvivor group size. Univariate logistic regression analysis was done to develop predictive models between scoring systems and mortality, with Hosmer-Lemeshow statistical analysis used to determine model goodness of fit.

Receiver operating characteristic (ROC) curve analysis was performed to evaluate the performance of scoring systems in predicting mortality. Following that, optimal cutoff point for each scoring system was identified by maximizing Youden's index. Last but not least, the corresponding accuracies, sensitivities, specificities, positive predictive values, and negative predictive values of the respective optimal cutoff points were calculated.

### 2.6. Patient Involvement

No patients were directly involved in this study. Hospitalization records were accessed anonymously, with only the relevant required information extracted for the study.

## 3. Results

A total of 152 patients aged 19 to 91 were identified in Linkou Chang Gung Memorial Hospital and Taipei Chang Gung Memorial Hospital over the span of 5 years. Statistically significant results (p < 0.05) are as follows, expressed in terms of survivors versus nonsurvivors: 0% versus 37.5% with terminal illness, pulse rate of 114 versus 144 beats per minute, respiratory rate of 20 versus 28 breaths per minute, 19.44% versus 75% with tachypnea or hypoxia, SpO_2_ of 100% versus 83%, mean arterial pressure of 76.67 mmHg versus 65.67 mmHg, 4.86% versus 25% with Glasgow Coma Scale <12 at presentation to ED, 9.72% versus 62.5% with altered mental status, 19.44% versus 62.5% with bandemia, 18.75% versus 87.5% with estimated glomerular filtration rate < 50%, 9.72% versus 62.5% with septic shock, MEDS score of 3 versus 12, REMS of 5 versus 9, RAPS of 2 versus 6, and MEWS of 4 versus 8 (Table 5).

Univariate logistic regression analysis of the scoring systems with respect to probability of death found the following odds ratios: 1.571 for MEDS (p = 0.0001), 1.529 for MEWS (p = 0.0035), 1.619 for RAPS (p = 0.0009), and 1.528 for REMS (p = 0.0009) (Table 6). Hosmer-Lemeshow statistical tests found all four models to be a good fit (MEDS 0.8249, MEWS 0.3598, RAPS 0.2430, and REMS 0.3111).

**Figure 1:**
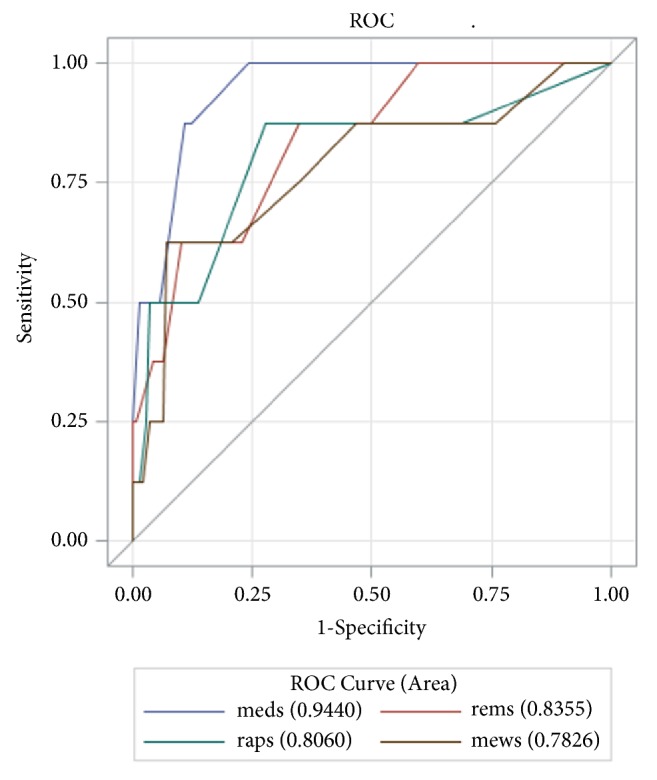
Receiver operating characteristic curves for predicting death according the RAPS, MEWS, REMS, and MEDS scoring systems.

Area under ROC (AUROC) analysis revealed the predictability of MEDS, REMS, RAPS, and MEWS as 0.9440 (p<0.001), 0.8355 (p=0.001), 0.8060 (p=0.004), and 0.7826 (p=0.007), respectively (Figure 1). With an optimal cutoff of 9, the negative predictive value of MEDS was found to be 99.22% (Table 7). Youden's indexes for the respective scores were as follows: MEDS 0.7639, REMS 0.5278, RAPS 0.5972, and MEWS 0.5556.

## 4. Discussion

This study is the largest yet examining renal abscess patients in the ED and also the first to the best of our knowledge to use ED physiologic scoring systems for risk stratification of these patients. We found MEDS to be the most ideal tool in predicting mortality rates of ED patients with renal abscess.

MEDS was designed and validated by Shapiro et al. in 2003 to risk stratify ED patients suspected to have infections according to risk of mortality. It comprises 9 parameters: age > 65 years, nursing home resident, rapid terminal comorbid illness, lower respiratory tract infection, bands >5% on a white blood cell count differential, tachypnea or hypoxemia, septic shock, platelet count < 150 x 10^9^/L, and altered mental status. The calculated score is then directly proportional to the patient's mortality risk [15]. Its original intended target population of ED patients with infections sets it apart from the other 3 scoring systems, which were designed with different patient populations in mind. This might explain why MEDS is the most accurate tool in predicting mortality of renal abscess patients, as these patients usually succumb to sepsis and septic shock.

MEDS has also been demonstrated to be a good predictor of prognosis in patients with other intra-abdominal infections [19, 21, 22] and even performs better than other more established risk assessment tools including APACHE II and quick Sepsis-related Organ Failure Assessment in determining mortality risk of ED patients in infection and/or sepsis [23–30]. Its superiority in this study provides further evidence that both clinical presentation and patient characteristics are significant in assessing mortality risk of patients with abdominal infections as mentioned in our introduction, almost half of the 9 criteria in MEDS (age, nursing home resident, terminal illness, altered mental status) are dependent on the patient's medical history that is easily and quickly ascertainable in the ED.

Univariate analysis in this study found that pulse rate, respiratory rate, tachypnea or hypoxia, SpO_2_, mean arterial pressure, Glasgow Coma Scale, altered mental status, estimated glomerular filtration rate, presence of septic shock, and positive history of terminal illness were significant in predicting the mortality of a renal abscess patient. These findings further reinforce the relationship between septic shock and mortality risk; other than presence of septic shock, most of the other significant parameters are classical findings in septic patients: tachycardia, tachypnea, hypotension, altered mental status, bandemia, and acute kidney injury. Patients with terminal illnesses are also more prone to developing sepsis. Interestingly, the number of abscesses did not have a significant impact on patient mortality, suggesting that preventing, limiting, and resolving sepsis are more important in mortality reduction than abscess characteristics.

The success of MEDS thus lies in its more suitable physiological parameters and incorporation of 5 significant variables (terminal illness, tachypnea or hypoxia, septic shock, bandemia, and altered mental status) into its scoring, enabling it to stratify patients with the highest accuracy rate of 88.82% and sensitivity of 87.50% amongst the 4 scoring systems studied. The high negative predictive value of 99.22% further enables emergency physicians and surgeons to decisively exclude renal abscess patients with a MEDS score of less than 9 from the high-risk group, and as such conservative management of antimicrobial therapy and/or percutaneous drainage can be justified, thus avoiding unnecessary emergency nephrectomies. The AUROC value of 0.9440 for MEDS also demonstrates that it is ideal for predicting mortality in patients with renal abscess. Though MEDS comprises more parameters than the other 3 physiologic scores, the extra information required is easily established through history taking and biochemical investigations routinely done during patient evaluation in the ED.

We nevertheless recognize that there will be certain situations in which patient history cannot be adequately established quickly in the ED, rendering emergency physicians unable to calculate MEDS. In these cases, our findings show that REMS is the next-best risk stratification tool to predict mortality in renal abscess patients, similar to study conclusions of our investigation into HPVG patients [18]. Future efforts could be directed into further determining the superiority of REMS in relation to RAPS and MEWS for ED populations with other abdominal infections, so as to identify the optimal score to employ if only patient's vital signs are available.

Despite being the largest study of renal abscess patients in the ED, this study is still limited by the small number of participants. There were only 2 patients who fulfilled each criteria of “nursing home resident” and “lower respiratory tract infection”, two important factors in the MEDS scoring system; the utility of MEDS in our study may therefore be an underestimate. Larger studies which include more patients who fulfil these criteria would aid in providing an even more accurate assessment of MEDS. Further studies to confirm these findings and prospectively validate the use of MEDS in this patient population is also required.

## 5. Conclusion

MEDS score is the best performing physiologic scoring system amongst the four studied systems in predicting the mortality of renal abscess patients. We recommend its employment in the ED for rapid risk stratification to promptly ascertain which patients require urgent intervention, thus ensuring timely and early intervention and subsequently improved patient outcomes.

## Figures and Tables

**Table 1:** Rapid Acute Physiology Score (RAPS) scoring system.

	**Score**				
**Variable**	0	+1	+2	+3	+4
**PR (/min)**	70–109		55–69	40–54	≤39
			110–139	140–179	≥180
**MAP (mmHg)**	70–109		50–69	130–159	≤49
			110–129		≥160
**RR (/min)**	12–24	10–11	6–9	35–49	≤5
		25–34			≥50
**GCS**	≥14	11–13	8–10	5–7	≤4

PR, pulse rate; MAP, mean arterial pressure; RR, respiratory rate; GCS, Glasgow Coma Scale.

**Table 2:** Rapid Emergency Medicine Score (REMS) scoring system.

	**Score**						
**Variable**	0	+1	+2	+3	+4	+5	+6
**Age (years)**	<45		45–54	55–64		65–74	>74
**PR (/min)**	70–109		55–69 110–139	40–54 140–179	≤39 >179		
**MAP (mmHg)**	70–109		50–69 110–129	130–159	≤49 >159		
**RR (/min)**	12–24	10–11 25–34	6–9	35–49	≤5 >49		
**GCS**	14 or 15	11–13	8–10	5–7	3 or 4		
**SpO**_**2**_ ** (%)**	>89	86–89		75–85	<75		

PR, pulse rate; MAP, mean arterial pressure; RR, respiratory rate; GCS, Glasgow Coma Scale; SpO_2_, peripheral oxygen saturation.

**Table 3:** Modified Early Warning Score (MEWS) scoring system.

	**Score**			
**Variable**	0	+1	+2	+3
**Systolic BP (mmHg)**	101–199	81–100	71–80 ≥200	<70
**Heart rate (/min)**	51–100	41–50 101–110	≤40 111–129	≥130
**Respiratory rate (/min)**	9–14	15–20	<9 21–29	≥30
**Temperature (**°**C)**	35–38.4		<35 ≥38.5	
**AVPU score**	**A**lert	Reacts to **V**oice	Reacts to **P**ain	**U**nresponsive

**Table 4:** Mortality in Emergency Department Sepsis (MEDS) scoring system.

**Variable**	**Points**
**Terminal illness** ^**1**^	6
**Age > 65 years**	3
**Tachypnea or hypoxia** ^**2**^	3
**Septic shock**	3
**Platelet count < 150 × 10** ^**9**^ **/L**	3
**Band > 5%**	3
**Lower respiratory infection**	2
**Nursing home resident**	2
**Altered mental status** ^**3**^	2

^1^Defined as disease condition with >50% likelihood of predicted fatality within 30 days or metastatic cancer. ^2^Defined as respiratory rate > 20 breaths/min *or* requiring oxygen by mask *or *SpO_2_ < 90%. ^3^Defined as any difference from the patient's baseline in any of the three spheres of orientation or in their level of alertness.

**Table 5:** Comparison of the characteristics of survivors and nonsurvivors.

**Variable**	**Patients**			
	**Total**	**Survivors**	**Non-survivors**	**p-value**
No.	152	144	8	
Age (years), Median (IQR)	54 (41-65)	54 (41-64)	67 (52-78.5)	0.082
Male, No. (%)	48 (31.58)	43 (29.86)	5 (62.5)	0.11
Terminal illness, No. (%)*∗*	3 (1.97)	0 (0.00)	3 (37.5)	<.001
Nursing home resident, No. (%)	2 (1.32)	2 (1.39)	0 (0)	1.000
Lower respiratory tract infection, No. (%)	2 (1.32)	1 (0.69)	1 (12.5)	0.103
Body temperature (°C), Median (IQR)	38.5 (37.55-39.45)	38.5 (37.6-39.45)	38.15 (36.15-39.35)	0.455
Pulse rate (/min), Median (IQR)*∗*	114 (102-127.5)	114 (101-125)	143.5 (115-156.5)	0.007
Respiratory rate (/min), Median (IQR)*∗*	20 (18-22)	20 (18-21)	27.5 (22.5-35)	0.001
Tachypnea or hypoxia, No. (%)*∗*	34 (22.37)	28 (19.44)	6 (75)	0.002
SpO_2_ (%), Median (IQR)*∗*	100 (95-100)	100 (96-100)	82.9 (65.65-92)	<.001
Mean arterial pressure (mmHg), Median (IQR)*∗*	75.33 (69-85)	76.67 (69.83-85.5)	65.67 (55-76.5)	0.021
Glasgow Coma Scale, No. (%)*∗*				0.034
≦8	2 (1.32)	1 (0.69)	1 (12.5)	
9~11	7 (4.61)	6 (4.17)	1 (12.5)	
≧12	143 (94.08)	137 (95.14)	6 (75)	
Altered mental status, No. (%)*∗*	19 (12.5)	14 (9.72)	5 (62.5)	<.001
AVPU score, No. (%)*∗*				0.003
A	137 (90.13)	133 (92.36)	4 (50)	
V	6 (3.95)	4 (2.78)	2 (25)	
P	7 (4.61)	6 (4.17)	1 (12.5)	
U	2 (1.32)	1 (0.69)	1 (12.5)	
Leukocyte count (/*μ*L), Median (IQR)	15.35 (11.3-20)	15.25 (11.3-19)	22.35 (6.4-27.05)	0.426
Platelets (×10^3^/*μ*L), Median (IQR)	222.5 (158-307)	223.5 (163-312.5)	153.5 (19-228.5)	0.081
Bandemia (Band>5%), No. (%)*∗*	33 (21.71)	28 (19.44)	5 (62.5)	0.013
eGFR, No. (%)*∗*				<.001
>50	118 (77.63)	117 (81.25)	1 (12.5)	
10-50	32 (21.05)	26 (18.06)	6 (75)	
<10	2 (1.32)	1 (0.69)	1 (12.5)	
Septic shock, No. (%)*∗*	19 (12.5)	14 (9.72)	5 (62.5)	<.001
Treatment, No. (%)				0.482
Antibiotics only	86 (56.58)	82 (56.94)	4 (50)	
Percutaneous drainage	58 (38.16)	54 (37.5)	4 (50)	
Surgical drainage	6 (3.95)	5 (3.47)	1 (12.5)	
Nephrectomy	4 (2.63)	4 (2.78)	0 (0.00)	
Numbers of abscess, No. (%)				0.178
Solitary	123 (80.92)	118 (81.94)	5 (62.5)	
Multiple	29 (19.08)	26 (18.06)	3 (37.5)	
Scoring system, Median (IQR)				
MEDS*∗*	3 (0-6)	3 (0-5)	11.5 (9-14.5)	<.001
REMS*∗*	5 (2-6.5)	4.5 (2-6)	9 (6-12.5)	0.001
RAPS*∗*	2 (0-4)	2 (0-4)	5.5 (4-7.5)	0.003
MEWS*∗*	4 (3-6)	4 (3-6)	8 (5.5-9)	0.007

*∗* indicates a statistically significant difference between survivors and nonsurvivors.

**Table 6:** Results of univariate logistic regression analysis for MEDS, MEWS, RAPS, and REMS with respect to probability of death.

**Variable**	OR (95% CI)	p-value
MEDS score*∗*	1.571 (1.249-1.977)	0.0001
MEWS score*∗*	1.529 (1.150-2.034)	0.0035
RAPS score*∗*	1.619 (1.220-2.149)	0.0009
REMS score*∗*	1.528 (1.189-1.962)	0.0009

*∗* indicates a statistically significant difference between survivors and nonsurvivors.

**Table 7:** Sensitivities, specificities, and accuracy rates of RAPS, MEWS, REMS, and MEDS for predicting mortality.

**Variable**	Accuracy rate	Optimal cut-off	Sen	Sp	PPV	NPV
MEDS	88.82%	9	87.50%	88.89%	30.43%	99.22%
REMS	66.45%	6	87.50%	65.28%	12.28%	98.95%
RAPS	73.03%	4	87.50%	72.22%	14.89%	99.05%
MEWS	91.45%	8	62.50%	93.06%	33.33%	97.81%

## Data Availability

The datasets used and/or analyzed during the current study are available from the corresponding author on reasonable request.
